# Health-Related Quality of Life of Diabetic Patients in Tehran

**DOI:** 10.5812/ijem.7945

**Published:** 2013-10-11

**Authors:** Ali Darvishpoor Kakhki, Zilla Abed saeedi

**Affiliations:** 1 Faculty of Nursing and Midwifery, Shahid Beheshti University of Medical Sciences, Tehran, IR Iran

**Keywords:** Diabetes, Health-Related Quality of Life, Health Status

## Abstract

**Background:**

Health-related quality of life (HRQoL) is an important factor for self-management behaviors of diabetic patients. These behaviors have special importance in preventing complications of diabetes.

**Objectives:**

This study has been conducted to evaluate HRQoL of diabetic patients referred to Tehran hospitals.

**Patients and Methods:**

In this descriptive study patients were selected from diabetes clinics of general hospitals in Tehran. A demographic and disease characteristics questionnaire and short-form of health survey (SF-36) were used for the data collection. The data were analyzed with SPSS software.

**Results:**

140 diabetic patients with average age of 47.3 ± 12.7 years participated in this study. The range of HRQoL scores in different domains varied from 46.2 ± 13 for general health perceptions to 64.1 ± 26.6 for physical functioning. There were significant differences according to age, sex, educational level, type of diabetes, type of treatment, and different HRQoL dimensions.

**Conclusions:**

HRQoL of diabetic patients is related to several variables. Considering of variables will be important for improving HRQoL of diabetic patients.

## 1. Background

Diabetes mellitus is one of the most prevalent chronic diseases in the world and the incidence of diabetes has increased dramatically in developing countries such as Iran (1-3). The acute and chronic complications of diabetes such as hypoglycemia, vascular complications, and renal complications are commonly diagnosed. In consequence of unsatisfactory control of diabetes, patients become increasingly subject to heart disease, blindness, sexual dysfunction and circulatory problems in addition to other complications (1, 4). Diabetes can also have considerable consequences for work, sexual activity, and leisure as well as for social and family life. In acute and chronic form debilitating and life threatening complications occur. The burden of disease management, complex and expensive therapeutic regimens, dietary restrictions, and the need to inject insulin and test blood and urine drastically impair quality of life (5, 6). The diagnosis of diabetes as with other major chronic illnesses, affects many aspects of an individual’s quality of life. Once the quality of life has been affected, self management, the adherence to therapeutic regimen and treatment success are in peril (7). Therefore, efforts to improve quality of life will lead to better management of the disease for a satisfactory outcome. The quality of life can be appropriately examined by subjective perceptions of patients using a quality of life questionnaire (8). Hence, this study reports the results of diabetic patient assessment in relation to their HRQoL and reveals the demographic variables associated with it. 

## 2. Objectives

This study was conducted to evaluate HRQoL of diabetic patients referred to Tehran hospitals.

## 3. Patients and Methods

This descriptive study was conducted on 140 diabetic patients who were referred to 4 general hospitals’ diabetic clinics in Tehran, between December 2009 and March 2010. These hospitals hold a diabetes clinic at least once a week. The researcher conducted the data collection in each person at least once a week. The inclusion criteria consisted of: 1) the ability to speak and understand Farsi (national language of Iran), 2) age between 18 to 65, 3) having been diagnosed with diabetes for more than 6 months, and 4) having no other comorbidity. The exclusion criteria were: 1) history of gestational diabetes and 2) inability to give informed consent. A questionnaire was used to collect data on the demographics and the disease characteristics. An Iranian version of the short-form health survey SF-36 was used (9). The participants responded to a self-administered questionnaire and provided demographic information on another questionnaire which included 9 items on age, gender, marital status, education level, onset of diabetes, type of diabetes treatment and recent history of hospitalization due to diabetes. The Iranian version of SF-36 (9) was used to determine HRQoL among the diabetic patients. This scale was developed in the United States with established validity and reliability among different groups of patients (10). The scale consists of 36 items with 8 subscales; physical functioning (10 items), role limitations due to physical problems (4 items), bodily pain (2 items), general health perceptions (5 items), vitality (4 items), social functioning (2 items), role limitations due to emotional problems (3 items) and perceived mental health (5 items). Also, the SF-36 has an item about health transition that is not part of any of the eight scales. Participant responses were coded, summed and transformed to a 0-100 scale, with higher scores indicating better physical and mental functioning and freedom from pain (11). 

### 3.1. Statistical Analysis

Data were analyzed by descriptive statistical tests (Spearman rank correlation coefficient and Mann Whitney, Kruskal Wallis, and LSD tests), using SPSS Version 13.

### 3.2. Ethical Considerations

Approval to conduct the study was confirmed by the Ethics Committee of Shahid Beheshti Medical University in Iran. All participants were assured of confidentiality, asked to sign an informed consent, and given informal instructions informing them they could refuse to answer any question or discontinue participation at any time. 

## 4. Results

The 140 diabetic patients who participated in this study responded to the disease and demographic characteristics questionnaires as indicated in Table 1. Participants’ overall scores on the SF-36 are displayed in Table 2. The lowest score was achieved in the general health perceptions scale (Mean = 46.2, SD = 12.94) and the highest score was attained for the physical functioning scale (Mean = 64.13, SD = 26.61). The scores of physical health component summery and mental health component summery were 56.64 (± 25.11) and 50.52 (± 14.17) respectively. 

**Table 1.** Demographic and Disease Characteristics of Participants

Variable	No. (%)	Mean ± SD
**Age, y**		47.3 ± 12.7
**Gender**		
Males	52 (39.7)	
Females	79 (60.3)	
**Education level**		
Primary School	47 (35.9)	
Secondary School	27 (20.6)	
High School	42 (32.1)	
University	15 (11.5)	
**Married Status**		
Single	16 (12.2)	
Married	105 (80.2)	
Divorced	3 (2.3)	
Widowed	7 (5.3)	
**Job Status**		
Employed	33 (25.2)	
Unemployed	72 (55)	
Retired	24 (18.3)	
Disabled	2 (1.5)	
**Duration of Diabetes, y**		8.83 ± 6.10
**Type of Diabetes**		
Type 1	20 (15.3)	
Type 2	111 (84.7)	
**Treatment**		
Diet therapy	4 (3.1)	
Insulin	35 (26.7)	
Oral Anti Hypoglycemic Agents	73 (55.7)	
Insulin & Oral Antihypoglycemic Agents	19 (14.5)	
**Any hospitalization related to diabetes in recent year**		
No	93 (71)	
One	26 (19.84)	
Two and more	12 (9.16)	

**Table 2.** Means for Eight Subscales of SF-36

Dimensions	Mean	SD	Median	Range
**Physical functioning**	64.13	26.61	65	95
**Role limitations due to physical problems**	50.58	36.11	50	100
**Bodily pain**	53.47	26.72	52	90
**General health perceptions**	46.20	12.94	45	75
**Vitality**	47.05	15.16	45	95
**Social functioning**	59.45	25.20	50	100
**Role limitations due to emotional problems**	49.61	38.67	66.66	100
**Mental health**	46.45	15.05	44	88

The SF-36 subscale scores were influenced by characteristic such as age, sex, education level, type of diabetes, and treatment of diabetes.

The subjects’ age was significantly associated with physical functioning and role limitations due to emotional problems. Older individuals reported worse physical functioning (r = -0.279) and more role limitations due to emotional problems (r= -0.235) than younger individuals (Table 3). Women attained significantly lower scores on role limitations due to physical problems and bodily pain than men (Table 4). There was a significant difference between education level and physical functioning (Table 4). The greatest difference was between individuals with primary school education versus university education (MD = 23.27) and then between individuals with secondary school education versus university education (MD = 17.92) and finally between individuals with high school education compared to university education (MD = 68.15). The type 2 diabetic patients attained significantly lower scores on physical functioning, role limitations due to physical problems, bodily pains and role limitations due to emotional problems than type 1 diabetic patients (Table 4). There were significant differences among treatment types and role limitations due to emotional limitations (Table 4). The greatest difference was found between the use of insulin versus oral anti-diabetic agents (MD= 36.70). Finally the greatest differences were found between those who used insulin and oral anti-diabetic agents (MD= 17.81) and between patients who used insulin and diet regimen (MD= -1.90). 

The SF-36 subscales scores were not significantly related to marital status, duration of diabetes and recent hospitalization due to diabetes.

**Table 3.** Correlation of Age and Eight Subscales of SF-36

Dimensions	R	P Value
**Physical functioning**	-0.279	0.002
**Role limitations due to physical problems**	-0.110	0.216
**Bodily pain**	-0.068	0.440
**General health perceptions**	-0.161	0.067
**Vitality**	-0.040	0.652
**Social functioning**	0.053	0.551
**Role limitations due to emotional problems**	-0.279	0.002
**Mental health**	-0.110	0.216

**Table 4.** Means and P Value for Eight Subscales of SF-36 According to Subgroups

	Physical Functioning	Role Limitations Due to Physical Problems	Bodily pain	General Health Perceptions	Vitality	Social Functioning	Role Limitations Due to Emotional Problems	Mental Health
**Gender**		P = 0.048	P = 0.035					
Males		58.33	59.52					
Females		45.51	49.48					
**Education level**	P = 0.041							
Primary School	57.80							
Secondary School	63.15							
High School	65.39							
University	81.07							
**Type of diabetes**	P = 0.002	P = 0.002	P = 0.027				P = 0.001	
Type 1	85.50	73.68	65.55				76.67	
Type 2	60.85	46.59	51.29				44.65	
**Treatment **							P = 0.005	
Diet therapy								
Insulin							64.76	
Oral Anti Hypoglycemic Agents							46.95	
Insulin & Oral Antihypoglycemic Agents		P = 0.048	P = 0.035				28.07	

## 5. Discussion 

The results revealed that diabetes has impact on HRQoL for diabetic patients at different dimensions. These findings were similar to other studies ( 12 , 13 ). Mean SF-36 scale scores for patients in this study ranged from 46.2 to 64.13 and were generally lower than similar studies ( 6 , 14 ). The relatively lower scores for these patients may indicate the impact of diabetes on HRQoL in Iran. The most notable effects were for general health perceptions and the least effect was for social functioning (Table 2). These findings are consistent with the results of other studies ( 6 , 14 ). The decrease in vitality, fatigue, depression, anger, and concerns about the disease complications and prognosis of diabetes were shared among the participants in this study ( 15 ). 

The significant negative correlations of age with physical functioning and role limitations due to emotional problems were among the findings. Physical problems are the most common complications for diabetic patients (16). Since age was associated with increased physical problems, it was viewed as having a synergistic effect on physical functioning of diabetic patients (17).

The diagnosis and management of diabetes could be perceived as a tension factor and lead to ineffective response by diabetic patients (18). On the other hand, aging accompanied added challenges such as financial demands of diabetes and worries about the patient and family futures (6). This finding showed the potential for decreased coping ability and role limitations due to emotional problems. Women showed significantly greater perceived impact of bodily pain and role limitations due to physical problems than men. In other studies also women attained lower quality of life and more problems than men (12, 17, 19). Probably, this is due to biological and psychological differences between men and women. Some physical functioning differences were found among levels of education. The patients with university education showed significantly better physical functioning than patients with less education. This is consistent with other studies (20-22). Probably, an increase in education leads to more flexibility in life and impetus for self-care that would lead to decrease in physical problems and improvement of physical functioning. On the other hand, education could leads to improvement in job status, and therefore social and economic situation, and consequently to well-being and access to health services. The scores obtained for physical functioning, role limitations due to physical problems, bodily pain, and role limitations due to emotional problems of patients with type 2 diabetes were lower than for patients with type 1 of diabetes. Although, patients with type 1 of diabetes in long term have more biological and physical complications than type 2 of diabetes (1), patients with type 1 of diabetes have more effective coping mechanisms than patients with type 2 of diabetes. This difference is also noted in similar studies (15). The results also indicated that there is a significant difference between the type of treatment regimen and role limitations due to emotional problems. The most difference was seen between insulin therapy and insulin therapy plus oral hypoglycemic agents and then between insulin therapy and oral hypoglycemic agents. The lowest difference was between insulin therapy and diet regimen. The patients with diet therapy usually have better quality of life than other therapeutic regimens (23, 24). These patients probably have better control and self care agency than patients with insulin or drug therapy. The lower referrals to physician and not having to use drug or insulin therapy which lead to more motivations, would reduce limitations due to emotional problems of patients. However, in other studies (14, 23) it has been indicated that patients with insulin therapy obtained lower scores than other regimen therapies but in this study patients with insulin therapy obtained a better score than those using other regimens. The study by Jamshidnia (22) also confirms the result of this study. However, findings here are different from studies by Johnson et al. (14) and Jacobson et al. (23) due to cultural influences regarding HRQoL in Iran. Participants in this study believed that using insulin therapy meant their diabetes status was worse. Therefore, patients who used insulin therapy had to have more motivation and less fear and anger compared to those who used other forms of treatments. Diabetes as with other diseases such as hemodialysis, can lead to decreased HRQoL. Planning and intervention are necessary to improve patients’ understanding of their diabetes and increase patient adherence to treatment.

 We acknowledge our study has some limitations. One is the modest sample size due to time and cost constraints. Hence, we interpret the results with caution. A second limitation is that the study has no control group. In addition to limitation in time and cost, it is difficult finding a suitable control group for quality of life as a subjective phenomenon (8). Thus, a comparison of the results of this study with a general population is not possible. 
